# Effect of Sows Gestational Methionine/Lysine Ratio on Maternal and Placental Hydrogen Sulfide Production

**DOI:** 10.3390/ani10020251

**Published:** 2020-02-05

**Authors:** Jie Peng, Mao Xia, Jia Xiong, Chenbin Cui, Ningning Huang, Yuanfei Zhou, Hongkui Wei, Jian Peng

**Affiliations:** 1 Innovative Institute of Animal Healthy Breeding, College of Animal Sciences and Technology, Zhongkai University of Agriculture and Engineering, Guangzhou 510225, China; pengjie@zhku.edu.cn; 2 Department of Animal Nutrition and Feed Science, College of Animal Science and Technology, Huazhong Agricultural University, Wuhan 430070, China; xiamao_1992@163.com (M.X.); xj511845341@126.com (J.X.); cuichenbin@webmail.hzau.edu.cn (C.C.); ninghuang2020@163.com (N.H.); zhouyuanfei@mail.hzau.edu.cn (Y.Z.); weihongkui@mail.hzau.edu.cn (H.W.); 3 The Cooperative Innovation Center for Sustainable Pig Production, Wuhan 430070, China

**Keywords:** hydrogen sulfide, reproductive performance, pregnant sows, methionine, placental angiogenesis

## Abstract

**Simple Summary:**

Hydrogen sulfide (H_2_S) is an important second messenger, which has been implicated in regulating placental angiogenesis. Our findings revealed that gestational dietary methionine could affect maternal and placental H_2_S concentrations. With the increase of dietary methionine, maternal plasma and placental H_2_S concentrations changed quadratically, which was consistent with placental vascular density and reproductive performance. The decrease in H_2_S production caused by an increase in dietary methionine was likely to be the cause for the increase in the rate of low birth weight piglets and needs further study.

**Abstract:**

The placenta is a unique bond between the mother and the fetus during pregnancy, and a proper placental angiogenesis is vital for fetal development. H_2_S is an endogenous stimulator of angiogenesis that is mainly produced by the methionine transsulfurationpathway. The goal of this study was to evaluate the effect of gestational dietary methionine on maternal and placental H_2_S production in sows. Multiparous sows (Large×White; third parity; *n* = 65) were randomly allocated into five groups, with feed diets comprisingstandardized ileal digestible methionine/lysine (Met/Lys) ratios of 0.27 (nutrient requirements of swine (NRC); 2012 level), 0.32, 0.37, 0.42, and 0.47, respectively. The litter size and weight at birth were measured and recorded. Maternal blood samples were obtained at embryonic day (E) E40 d, E90 d, and E114 d of gestation. The placental samples were collected at parturition. The results showed that maternal plasma H_2_S concentration was not affected at E40 d. However, the maternal plasma H_2_S concentration changed quadratically with the dietary Met/Lys ratio at E90 d (*p* < 0.01) and E114 d (*p* = 0.03). The maximum maternal plasma H_2_S concentration was at the dietary Met/Lys ratio of 0.37. Meanwhile, maternal plasma H_2_S concentration was positively correlated with piglets born alive (*p* < 0.01) and litter weight (*p* < 0.01). Consistent with the maternal plasma, the placental H_2_S concentration also changed quadratically with the dietary Met/Lys ratio (*p* = 0.03); the Met/Lys ratio of 0.37 showed the maximum H_2_S concentration. In conclusion, our findings revealed that the gestational dietary Met/Lys ratio could affect maternal and placental H_2_S concentrations, which may be an important molecular mechanism affecting placental angiogenesis and piglet development.

## 1. Introduction

Methionine (Met) is an essential amino acid in the diets of pigs [1,2]. In addition to being a substrate for protein synthesis, Met is also a methyldonor in DNA, RNA, and protein methylation. This methylation reaction results in epigenetic changes in gene expression and protein function during embryonic development [3,4]. Functional amino acids are important for the development of the pig placenta [5]. In our previous work, we demonstrated that an appropriate increase in the methionine/lysine (Met/Lys) ratio in a sow’s diet had a positive effect on the sow’splacental vascular density and on the birth weight of the piglets [6]. However, the underlying mechanism by which Met promotes placental angiogenesis and fetal development is unclear. 

Hydrogen sulfide (H_2_S) is a ubiquitous signaling molecule with important functions in many mammalian cells and tissues and is now considered to be the third gas-phase signaling molecule, in addition to carbon monoxide (CO) and nitric oxide (NO) [7,8,9]. Endogenous H_2_S is mainly produced by Met and the cysteine transsulfuration pathway (TSP), via the direct enzymatic action of cystathionine-γ-lyase (CGL) and cystathionine-β-synthase (CBS) [10,11]. Studies in humans have found that preeclampsia and intrauterine growth retardation (IUGR) correspond to a significant reduction in placental H_2_S production, placental angiogenesis, and blood flow velocity [12,13]. However, exogenous H_2_S donors significantly attenuated placental vascular dysplasia and IUGR, caused by CGL inhibition [14]. In vitro studies have also shown that H_2_S produced by trophoblast cells can promote angiogenesis in placental artery endothelial cells [15]. However, there were no reports of the regulation of H_2_S production in sow plasma and placenta. The goal of the current study was to investigate the role of the gestational methionine/lysine ratio on maternal and placental H_2_S production, both in vivo and in vitro. 

## 2. Materials and Methods 

### 2.1. Animals, Diets and Management 

The experimental design and the dietary formula were described previously [6]. In this study, multiparous sows (Large×White; third parity; *n* = 65) were randomly allocated into five groups and fed diets with standardized ileal digestible methionine/lysine (Met/Lys) ratios of 0.27 (nutrient requirements of swine (NRC); 2012 level), 0.32, 0.37, 0.42, and 0.47 respectively. The diet was maintained at constant levels of Lys (average SID Lys 0.71%); the ingredients and nutrient compositions of the experimentaldiets are listed in Table S1. Sows were fed a gestational dietary 2.0 kg/day (from embryonic day E0 d to E3 d), 2.8 kg/day (E4 d to E30 d), 2.3 kg/day (E31 d to E90 d), and 2.9 kg/day (E91 d to farrowing). All sows were moved to individual farrowing crates with stalls at E90 d. The experimental protocol was approved by the Institutional Animal Ethics Committee of Huazhong Agricultural University(Ethics Code: HZAU SW-2016-014).

### 2.2. Recording and Sampling

The sample collection of blood and placenta was described previously [6]. Twelve sows by group were selected for blood sampling, and eight sows of each group were selected for placenta sampling. The litter size, the piglets born alive, the litter weight, and the average pig birth weight of each litter were recorded within 24 h after farrowing.

### 2.3. Preparation of Placenta Homogenate

The placenta was selected based on the average pig birth weight ± one standard deviation of each group. A total of 100 mg of placental tissue was separated and ground to a fine powder using a porcelain mortar and pestle, chilled with liquid nitrogen. Next, 10% of the tissue homogenate was made with 100 mM of ice cold KH_2_PO_4_ buffer (pH 7.4) and was centrifuged for 5 min at 13,000 rpm. The supernatant wasused for H_2_S content and production assay. The protein concentration was determined by the bicinchoninic acid (BCA) protein assay kit (Beyotime, Shanghai, China).

### 2.4. Cell Culture and Treatment

Porcine iliac artery endothelial cells (PIEC) were obtained from the cell bank of the Chinese Academy of Sciences (Shanghai, China), cultured in Roswell Park memorial institute (RPMI) 1640 medium (Gibco, San Diego, CA, USA), and supplemented with 10% fetal bovine serum (FBS, Gibco, San Diego, CA, USA) and 1% penicillin/streptomycin solution (Sigma, St. Louis, MO, USA) at 37 °C in a 5% CO_2_ incubator. Before the Met treatment, the PIEC cells were seeded in a 12-well plate (2 × 10^5^ cells/well); 80% confluent PIEC cells were placed in serum-free basic medium for 18 h, followed by Met-free RPMI 1640 medium for another 6 h, then treated with different concentrations of Met (0, 50, 200, 500, and 1000 μM) for 24 h (*n* = 4). The supernatant culture medium was collected for H_2_S content assay.

### 2.5. H_2_S Measurement

In this study, the H_2_S levels were measured through the formation of methylene blue [16,17]. For the plasma, 100 μL samples were mixed with 400 μL of 100 mM KH_2_PO_4_ buffer (pH 7.4) and 250 μL of 1% (w/v) zinc acetate; for the culture medium and placental tissue homogenate, 500 μL samples were mixed with 250 μL of 1% (w/v) zinc acetate. The reaction mixture was incubated at 37 °C for 2.5 h. The protein content in the samples was removed by adding 10% trichloroacetic acid (250 μL) to the reaction mixture, which was pelleted by centrifugation. The supernatant was then mixed with 100 μL of *N*-dimethyl-p-phenylenediamine sulfate (20 mM in 7.2 M HCl) and 100 μL of FeCl_3_ (30 mM in 1.2 M HCl) in a test tube. The mixture was incubated at room temperature for 15 min. The absorbance of the resulting solution was measured at 670 nm in a 96-well plate with a microplate reader. The H_2_S concentration was calculated using a standard (NaHS) calibration curve (0.04, 0.2, 0.4, 2, 4, 20, and 40 μM) (Aladdin, Shanghai, China).

### 2.6. Statistical Analyses

**Figure 1:**
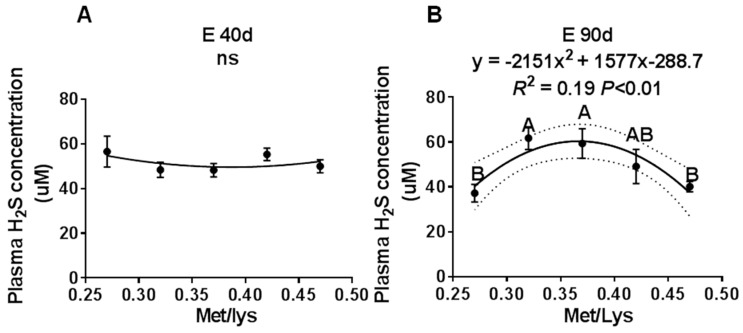

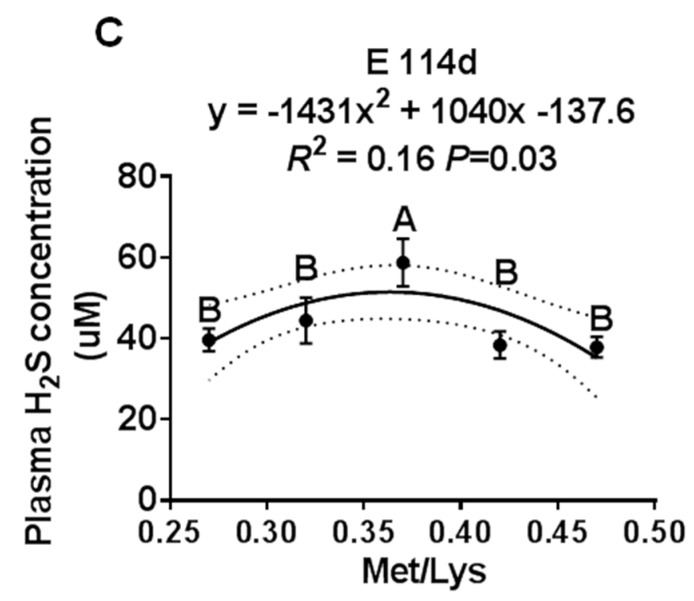
The effect of the gestational dietary Met/lysine ratio on maternal plasma hydrogen sulfide (H_2_S) concentration at E40, E90, and E114: (**A**) maternal plasma H_2_S concentration at E40 d, (**B**) maternal plasma H_2_S concentration at E90 d, and (**C**) maternal plasma H_2_S concentration at E114 d, *n* = 8–12/group.

**Figure 2:**
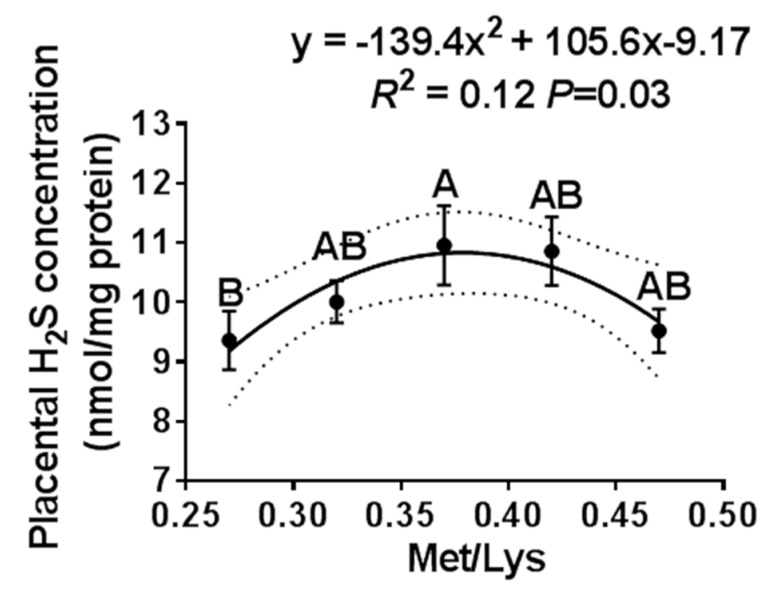
The effect of the gestational dietary Met/lysine ratio on placental H_2_S concentration. Met/Lys ratios 0.27, *n* = 12; Met/Lys ratios 0.32, *n* = 14; Met/Lys ratios 0.37, *n* = 11; Met/Lys ratios 0.42, *n* = 11; and Met/Lys ratios 0.47, *n* = 11.

**Figure 3:**
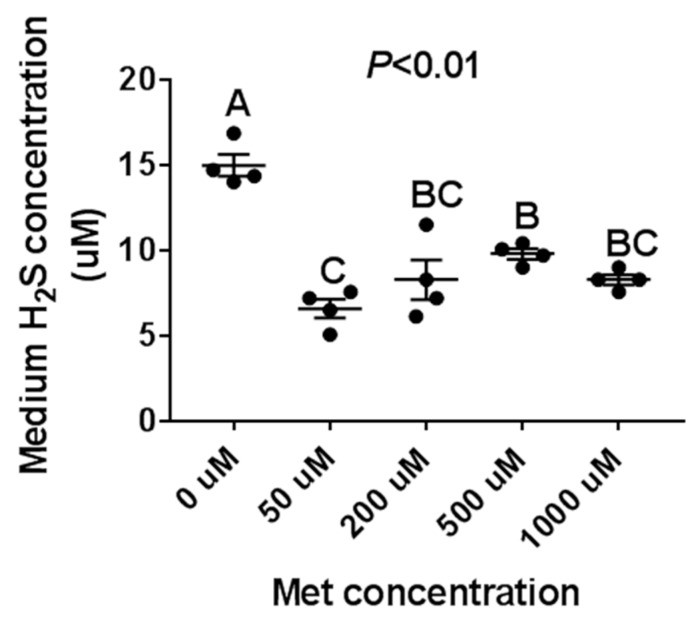
The effect of the Met treatment on porcine iliac artery endothelial cells (PIEC) H_2_S production. The PIEC cells were seeded in a 12-well cell plate. After 80% confluence, the PIEC cells were placed in a serum-free medium for 18 h, followed by a Met-free RPMI 1640 medium for another 6 h, then treated with Met for 24 h. The supernatant culture medium was collected for H_2_S assay, *n* = 4/group.

**Figure 4:**
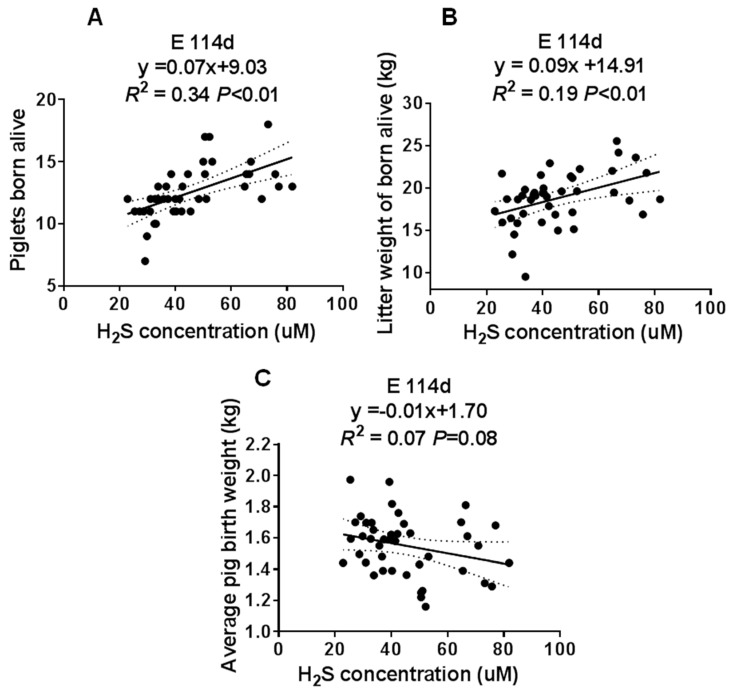
The correlation analysis of the maternal plasma H_2_S concentration at E114d and the sow’s reproductive performance. (**A**) Piglets born alive. (**B**) Litter weight of piglets born alive. (**C**) Average pig birth weight. Met/Lys ratios 0.27, *n* = 8; Met/Lys ratios 0.32, *n* = 9; Met/Lys ratios 0.37, *n* = 10; Met/Lys ratios 0.42, *n* = 7; Met/Lys ratios 0.47, *n* = 7.

Statistical analysis was performed using the SAS statistical package (v 8.2; SAS Inst. Inc., Cary, NC, USA). A one-way analysis of variance (ANOVA) followed by a least-significant-difference (LSD) post-hoc test was conducted to explore the impact of the gestational dietary Met/lysine ratio on maternal plasma and placenta H_2_S concentrations, as shown in Figure 1, Figure 2 and Figure 3, where *p* < 0.05 was considered to indicate statistical significance. Regression analyses were performed to evaluate the quadratic effects of dietary Met/Lys ratio and H_2_S concentration, as shown in Figure 1 and Figure 2; the linear effects of dietary Met/Lys ratio and H_2_S concentration are shown in Figure 4. Spearman correlations were used to determine the association between the dietary Met/Lys ratio and the piglets born alive, the litter weight of those born alive, and the average pig birth weight; see Figure 4. Data in all the Figures are expressed as mean ± standard errors of the mean (SEM).

## 3. Results

### 3.1. The Gestational Dietary Met/Lys Ratio Affected Maternal Plasma H_2_S Concentration

To study the effect of the gestation dietary Met/Lys ratio on maternal plasma H_2_S concentration, we collected maternal plasma at different stages of pregnancy. We found that by E40 d, increasing the Met/Lys ratio in the diet had no effect on the maternal plasma H_2_S concentration, as shown in Figure 1A. However, at E90 d and E114 d, the maternal plasma H_2_S concentration changed quadratically, with an increase in the dietary Met/Lys ratio, as shown in Figure 1B,C. According to the quadratic polynomial regression equation, at E90 d (y = −2151x^2^ + 1577x −288.7, R^2^ = 0.19, *p* < 0.01) and at E114 d (y = −1431x^2^ + 1040x −137.6, R^2^ = 0.16, *p* = 0.03), the maximum maternal plasma H_2_S concentration was at the dietary Met/Lys ratio of 0.37. These results indicated that maternal plasma H_2_S concentration could be affected by the dietary Met/Lys ratio.

### 3.2. Maternal Plasma H_2_S Concentration Was Positively Correlated with the Sows’ reproductive performance

To determine the involvement of H_2_S in the reproductive performance of the sows, we analyzed the correlation between the maternal plasma H_2_S concentration and the number of piglets born alive, the litter weight, and average pig birth weight at E114 d. We found that the maternal plasma H_2_S concentration had a positive correlation with the number of piglets born alive and the litter weight, as shown in Figure 4A,B. However, there was no correlation with the average pig birth weight, as shown in Figure 4C. These data suggested that maternal plasma H_2_S may have played an important role in the sows’ reproductive performance.

### 3.3. The Gestational Dietary Met/Lys Ratio Affected Placental H_2_S Concentration

We further examined the effect of the gestation dietary Met/Lys ratio on the placental H_2_S concentration. The placenta was selected based on the average pig birth weight ± one standard deviation of each group. Similar to the maternal plasma, the placental H_2_S concentration also changed quadratically with the dietary Met/Lys ratio, as shown in Figure 2. According to the quadratic polynomial regression equation (y = −139.4x^2^ + 105.6x −9.17, R^2^ = 0.12, *p* = 0.03), the maximum placental H_2_S concentration was at the dietary Met/Lys ratio of 0.37.

### 3.4. The H_2_S Productionof Met Treatment Affected PIEC Cells

To further confirm the effect of Met on H_2_S production in vitro, PIEC cells were treated with variable mediums which were (1) Met-free (0 μM), (2) had a physiological concentration of Met (50 μM), and (3) had a high concentration of Met (200, 500, and 1000 μM), respectively. We found that the H_2_S concentration was highest in the Met-free medium condition and lowest in a physiological concentration of Met (50 μM), as shown in Figure 3. Subsequently, with an increase in Met concentration, the H_2_S production increased initially (from 50 to 500 μM) and then maintained relative stability (from 500 to 1000 μM).

## 4. Discussion

H_2_S is an endogenous stimulator of angiogenesis that promotes vascular endothelial cell proliferation, migration, and angiogenesis by activating the NO/cGMP pathway, opening K_ATP_ channels, and promoting the phosphorylation of PI3K/AKT and MAPK pathways [7,18,19]. In the present study, we found that the maternal plasma H_2_S concentration was positively correlated with the sows’ reproductive performance. With an increase in Met/Lys ratio in the sows’ diet, the maternal plasma and the placental H_2_S concentrations changed quadratically. These results indicated that there was a dose-effect of Met concentration on H_2_S production.

Although Met is a precursor for H_2_S production, recent studies have found that dietary restriction (50% restriction) or Met restriction is the most effective way to increase H_2_S production. Studies have found that limiting the feed intake can increase liver CGL expression, increase endogenous H_2_S production, and alleviate liver ischemia-reperfusion injury [11]. Additionally, Met restriction also promotes vascular regeneration and recovery after rodent femoral artery ligation [20] and maintains capillary density in skeletal muscle [21]. Recently, two important studies have further confirmed that by restricting the sulfur amino acid, H_2_S production can increase in the skeletal muscles by up-regulating the GCN2/ATF4 pathway and activating NAD+/SIRT1 signaling, thus triggering skeletal muscle angiogenesis and delaying muscle aging [22,23]. We found similar results in our study, where medium H_2_S concentrations were highest under Met-free conditions (0 μM), whereas relative to physiological concentrations, high concentrations of Met did not significantly inhibit H_2_S production in PIEC cells, which suggested that there were some differences between in vitro and in vivo experiments.

In modern animal production, gestational restriction feeding is an effective and widely used practice to improve the reproductive performance of sows. Our previous study found that under the restriction feeding conditions, increasing the Met/Lys ratio from 0.27 to 0.37 can significantly increase the average pig birth weight and the litter weight in the pigs that are born alive [6]. However, when Met/Lys ratio was further increased to 0.47, the rate of piglets with a weight <0.9kg increased and the placental vascular density decreased significantly [6]. In this study, the maternal plasma at E114 d and the placental H_2_S concentrations were consistent with the sows’ reproductive performance. The decrease in H_2_S production caused by an increase in dietary Met was likely to be the cause for the increase in the rate of low birth weight, along with the failure to further improve the reproductive performance of the sows. Studies of rodents have found that a high concentration of Met can significantly inhibit CGL activity in peritoneal macrophages, reducing serum H_2_S content and increasing inflammatory reactions [16]. A recent study showed that exogenous H_2_S could promote mammary epithelial cell proliferation by activating the PI3K/Akt-mTOR pathway in porcine [24]. Therefore, determination of the appropriate dietary Met content is important for improving the reproductive performance of sows. Meanwhile, increasing the activity of maternal and placenta H_2_S producing enzymes, or increasing the H_2_S content by using H_2_S donors, may be an effective way to alleviate the adverse effects of high Met production. Our current results found that H_2_S donors diallyl trisufide(DATs) could significantly promote PIEC angiogenesis (unpublished data), which suggests that H_2_S is a potential target for improving the reproductive performance of sows.

## 5. Conclusions

In conclusion, our findings revealed that gestational dietary Met could affect maternal and placental H_2_S concentration, which may be an important molecular mechanism affecting placental angiogenesis and piglet development.

## Supplementary Materials

The following are available online at https://www.mdpi.com/2076-2615/10/2/251/s1, Table S1: Ingredients and nutrient compositions of the experimental diets.
