# Supplementary material for: Effect of Sows Gestational Methionine/Lysine Ratio on Maternal and Placental Hydrogen Sulfide Production
Source: Animals (Basel). 2020 Feb 5;10(2):251. doi: 10.3390/ani10020251 (PMC7070849; doi:10.3390/ani10020251)
Supplement: Supplementary file 1 [file animals-10-00251-s001.pdf]

animals

Supplementary Files: Effect of Sows Gestational Methionine/Lysine Ratio on Maternal and Placental Hydrogen Sulfide Production

Jie Peng [1,2,+], Mao Xia [2,+],Jia Xiong [2],Chenbin Cui [2], Ningning Huang [2], Yuanfei Zhou [2],Hongkui Wei [2] and Jian Peng [2,3,*]

Table S1. Ingredients and nutrient compositions of the experimental diets.

Item	Met/Lys=0.27	Met/Lys=0.32	Met/Lys=0.37	Met/Lys=0.42	Met/Lys=0.47
			Feedstuff%		
Corn	10.00	10.00	10.00	10.00	10.00
Sorghum	24.54	24.54	24.54	24.54	24.54
Barley	37.89	37.89	37.89	37.89	37.89
Soybeanhulls	2.00	2.00	2.00	2.00	2.00
Ricebran	7.50	7.50	7.50	7.50	7.50
Soybeanmeal	9.35	9.35	9.35	9.35	9.35
Palmmeal	5.00	5.00	5.00	5.00	5.00
98%Lysine	0.28	0.28	0.28	0.28	0.28
DL-methionine	0.00	0.04	0.07	0.11	0.14
L-threonine	0.10	0.10	0.10	0.10	0.10
Limestone	1.44	1.43	1.42	1.42	1.40
Calciumphosphate (monocalcium)	0.89	0.88	0.87	0.84	0.83
Sodiumchloride	0.47	0.46	0.44	0.44	0.43
Cholinechloride	0.14	0.14	0.14	0.14	0.14
Premix[1]	0.40	0.40	0.40	0.40	0.40
Total%	100.00	100.00	100.00	100.00	100.00
			Composition (calculated)[1]		
NEkcal/kg	2317.25	2318.70	2320.15	2321.60	2323.04
CP%	13.92	13.94	13.96	13.98	14.00
SIDLys%	0.71	0.71	0.71	0.71	0.71
SIDMet%	0.19	0.23	0.26	0.30	0.33
SIDThr%	0.48	0.48	0.48	0.48	0.48
SIDTrp%	0.14	0.14	0.14	0.14	0.14
Met/Lys	0.27	0.32	0.37	0.42	0.47
CF%	3.57	3.57	3.57	3.57	3.57
NDF%	14.53	14.53	14.53	14.53	14.53
ADF%	7.78	7.78	7.78	7.78	7.78
Starch%	44.74	44.74	44.74	44.74	44.74
Ash%	6.03	5.99	5.96	5.92	5.89
Ca%	1.22	1.21	1.21	1.20	1.20
P%	1.05	1.05	1.04	1.04	1.03

[1]Calculated chemical concentrations using values for feed ingredients from NRC (2012) [1].

References

1. National Research Council. *Nutrient Requirements of Swine*, 11th Edition; The National Academies Press and the Transportation Research Board: Washington, D.C., USA, 2012, pp. 44.
